# Genomic and single cell sequencing facilitate the dissection of heterogeneity of pancreatic tumors

**DOI:** 10.1186/s12916-020-01637-3

**Published:** 2020-07-08

**Authors:** Barish H. Edil, Wenyi Luo, Min Li

**Affiliations:** 1 Department of Surgery, The University of Oklahoma Health Sciences Center, Oklahoma City, OK 73104 USA; 2 Department of Pathology, The University of Oklahoma Health Sciences Center, Oklahoma City, OK 73104 USA; 3 Department of Medicine, The University of Oklahoma Health Sciences Center, 975 NE 10th Street, BRC 1262A, Oklahoma City, OK 73104 USA

**Keywords:** Pancreatic ductal adenocarcinoma, PanNET, Single-cell sequencing, Tumor heterogeneity

## Background

Multi-omics sequencing techniques have been applied to dissect inter- and intra-tumoral heterogeneity. Two recent studies from Dr. Wenming Wu’s group at the Department of General Surgery, Peking Union Medical College Hospital (PUMCH), applied single-cell RNA sequencing and whole-genome sequencing (WGS) to delineate heterogeneity of pancreatic tumors, including pancreatic ductal adenocarcinoma (PDAC) and pancreatic neuroendocrine tumor (PanNET). These works provide valuable resources for translational use in target therapy and prognostic prediction [1, 2]. Of note, PUMCH is a high-volume institute with enriched experience in pancreatic tumor treatment [3] with full spectrums of surgical techniques that include open, laparoscopic, and robotic surgeries [4, 5]. It is also a leading center of multi-center studies for pancreatic tumors [6].

## Tumor heterogeneity in PDAC

PDAC is characterized by a high degree of intra-tumoral heterogeneity. On average, the stroma constitutes over 70% of the tumor mass. To facilitate providing therapeutic targets and novel prognosis markers, characterization of each cell component and the associated critical factors in regulating PDAC progression is needed. Single-cell sequencing is pivotal for exploring tumor heterogeneity and dissecting the tumor-related mechanism in detail. In a recent paper published in *Cell Research* [1], Peng et al. applied single-cell RNA sequencing to 24 primary PDAC tumors and 11 control pancreases. Transcriptomic profiles of 57,530 cells were acquired. This study identified ten types of cells and characterized the features of gene expression profiles in each cell type of PDAC samples. Interestingly, two types of ductal cells were identified; the type II ductal cells expanded in PDAC are malignant. These malignant cells were further divided into distinct subgroups, and a proliferative subgroup was identified. This finding appears to be clinically relevant. First, the patients with more abundant proliferative ductal markers displayed significantly lower survival. Second, CDK1, PLK1, and AURKA were identified and verified to serve as pharmacological targets of proliferative ductal cells. Moreover, less infiltration and inactivation of T cells in PDAC patients were related to a high abundance of proliferative ductal markers. Thus, both the existence of proliferative ductal cells and the loss of T cell activation probably contribute to the poor prognosis of PDAC patients. In addition, heterogeneity of the tumor microenvironment was explored, and distinct subtypes of immune cells and fibroblasts in PDAC were characterized.

The seminal work by Peng et al. shed new light on the translational study of PDAC. This elegant study provided valuable resources for deciphering heterogenous cell types in PDAC. Exploring highly expressed pathways in stromal components, such as fibroblasts, will delineate the underlying mechanisms that are pivotal for tumor microenvironment maintenance.

This work built on prior studies to reveal potential prognostic markers for PDAC. Proliferative markers were used to cluster the Cancer Genome Atlas Program (TCGA) PDAC patients. The distinct survival rate was related to the abundance of markers. T cell infiltration status and T cell characteristics are usually associated with different prognostic outcomes [7]. Peng et al. also revealed that inactivation of T cells in PDAC patients is related to a high abundance of proliferative ductal markers. This finding provides new insight into understanding how distinct tumor types regulate their immune heterogeneity.

Beyond these biomarker-related and mechanistic aspects, this work also sheds new light on delineating the potential therapeutic targets of PDAC. From one side, malignant ductal cells can be divided into seven distinct subgroups. Based on the analysis of dysregulated pathways of the proliferative subgroup, Peng et al. identified pharmacological targets of proliferative ductal cells. For this, it may be important to characterize other subtypes, such as the subtype expressing high invasive markers, to further develop new targets of PDAC. From another side, the observation of T cell inactivation related to the high abundance of proliferative ductal markers in PDAC patients suggests a potential new combination strategy comprising cell cycle inhibitors and immunotherapies.

## Tumor heterogeneity in PanNETs

The other original article published in *GUT* [2] revealed genomic inter-tumoral heterogeneity of PanNETs. Clinical heterogeneity is well-known for the differences in hormone-related symptoms. Therefore, PanNETs could be mainly categorized as functional and non-functional PanNETs (NF-PanNETs). The current study systematically compared the genomic alterations of insulinoma (the major type of functional PanNETs) (*n* = 84) and NF-PanNETs (*n* = 127) with a combined cohort of 211 patients from PUMCH and the International Cancer Genome Consortium (ICGC).

Over the past decade, previous sequencing studies have revealed the genetic background of PDAC. Two major advances of PanNET sequencing were made. In 2011, WES uncovered dominant mutations [8]. In 2017, WGS revealed the genomic landscape [9]. However, those studies were conducted predominantly with NF-PanNETs, and the heterogeneity between insulinomas and NF-PanNETs has not been fully studied. The current study filled the gaps by revealing the genomic difference between insulinomas and NF-PanNETs. A new molecular classification system that is mainly based on a copy number variation (CNV) pattern has been proposed. NF-PanNETs have CNV deletions, copy neutral, and CNV amplification subtypes. However, insulinomas only have copy neutral and CNV amplification subtypes and lack the CNV deletion subtype.

PanNETs are also marked by heterogeneous clinical relapse risk. Insulinomas have less relapse risk than do NF-PanNETs. In NF-PanNETs, different individuals have different risks of relapse. Previous studies focused on *DAXX/ATRX* mutations for relapse/recurrence risk stratifications [10]. The current study provided a more precise understanding of *DAXX/ATRX* mutations and CNV patterns in NF-PanNETs. However, CNV patterns of *DAXX/ATRX* wild-type patients include both CNV amplifications/deletions and the copy neutral subtype. As for relapse risk, either CNV amplifications/deletions or *DAXX/ATRX* mutation predict worse prognoses. In addition, the combined use of CNV amplifications/deletions and *DAXX/ATRX* mutation provided a more precise prediction of relapse risks in a 2- and 5-year follow-up period. This result is an example of how heterogeneous clinical outcomes could be explained by underlying genetic differences.

## Conclusions

In summary, these studies by Peng et al. and Hong et al. applied different sequencing techniques to delineate intra-heterogeneity of PDAC and inter-heterogeneity of PNET. They mapped the cell atlas of PDAC, which led to a new classification system of PNET. These seminal works open a large panel of perspectives to facilitate precise medicine in pancreatic tumors.

## Data Availability

Not applicable

## Abbreviations

WGS: Whole-genome sequencing
PDAC: Pancreatic ductal adenocarcinoma
PanNET: Pancreatic neuroendocrine tumor
CNV: Copy number variation
